# Mice and Rats Display Different Ventilatory, Hematological, and Metabolic Features of Acclimatization to Hypoxia

**DOI:** 10.3389/fphys.2021.647822

**Published:** 2021-03-12

**Authors:** Christian Arias-Reyes, Jorge Soliz, Vincent Joseph

**Affiliations:** Centre de Recherche de l’Institute Universitaire de Cardiologie et de Pneumologie de Québec, Université Laval, Québec, QC, Canada

**Keywords:** acclimatization to hypoxia, physiology, metabolism, mitochondria, rodents

## Abstract

Phylogeographic studies showed that house mice (*Mus musculus*) originated in the Himalayan region, while common rats (*Rattus rattus* and *Rattus norvegicus*) come from the lowlands of China and India. Accordingly, it has been proposed that its origins gave mice, but not rats, the ability to invade ecological niches at high altitudes (pre-adaptation). This proposal is strongly supported by the fact that house mice are distributed throughout the world, while common rats are practically absent above 2,500 m. Considering that the ability of mammals to colonize high-altitude environments (>2,500 m) is limited by their capability to tolerate reduced oxygen availability, in this work, we hypothesize that divergences in the ventilatory, hematological, and metabolic phenotypes of mice and rats establish during the process of acclimatization to hypoxia (Hx). To test this hypothesis male FVB mice and Sprague-Dawley (SD) rats were exposed to Hx (12% O_2_) for 0 h (normoxic controls), 6 h, 1, 7, and 21 days. We assessed changes in ventilatory [minute ventilation (V_E_), respiratory frequency (*f*_R_), and tidal volume (V_T_)], hematological (hematocrit and hemoglobin concentration), and metabolic [whole-body O_2_ consumption (VO_2_) and CO_2_ production (VCO_2_), and liver mitochondrial oxygen consumption rate (OCR) parameters]. Compared to rats, results in mice show increased ventilatory, metabolic, and mitochondrial response. In contrast, rats showed quicker and higher hematological response than mice and only minor ventilatory and metabolic adjustments. Our findings may explain, at least in part, why mice, but not rats, were able to colonize high-altitude habitats.

## Introduction

Animals living at high-altitude present a series of physiological adjustments to prevent an imbalance in the supply and demand of oxygen (Scott et al., 2008; Storz et al., 2010). These adjustments occur along the oxygen cascade, and include a combination of physiological, cellular, molecular, and biochemical modifications that result in either an increased supply, narrowed demand, more efficient use of oxygen, or combinations of those three (Hochachka, 1985; Scott et al., 2008; Storz et al., 2010). Remarkably, such changes may differ in expression and efficiency between species or even between populations of the same species depending on their genetic backgrounds and their phenotypic plasticity (Monge and León-Velarde, 2003; Storz et al., 2010; Storz and Scott, 2021). Such differences appear when comparing house mice (*Mus musculus*), that successfully colonized high-altitude environments, and common rats (*Rattus rattus* and *Rattus norvegicus*), that are rare or absent in natural habitats over 2,500 m above sea level (masl; Jochmans-Lemoine et al., 2015). In previous works, we described the physiological traits of lab mice (FVB) and Sprague-Dawley (SD) rats established at 3,600 masl for more than 20 years and over 30 generations. Compared to rats, mice have a higher metabolic rate, higher tidal volume, increased alveolar surface area and lung volume, lower hematocrit and hemoglobin levels, and reduced right ventricular hypertrophy (a sign of reduced pulmonary hypertension; Jochmans-Lemoine et al., 2015). In following, we were interested in unveiling whether these features had a genetic basis or were the result of phenotypic plasticity, thus, the physiological and molecular responses to acute hypoxia (Hx; 6 h – 12% O_2_, roughly equivalent to 4,000 masl) were evaluated at sea level. In comparison to rats, mice had lower metabolic rate, higher minute ventilation, and higher expression of the transcription factor HIF-1α (a master regulator of the hypoxic response) in the brainstem. On the other hand, rats presented increased expressions of glucose transporter 1, indicating an elevated glycolytic metabolism in the brainstem (Jochmans-Lemoine et al., 2016). In summary, these data showed that FVB mice develop a more efficient response than SD rats during the acute phase of exposure to Hx, but it remains unclear whether such differences would persist during acclimatization to chronic hypoxia, a process of gradual changes occurring within few weeks that improves the capture, distribution, and utilization of oxygen (Monge and Leon-Velarde, 1991; Schmitt et al., 1994; Cáceda et al., 2001; Storz et al., 2010; Czerniczyniec et al., 2015; Lukyanova and Kirova, 2015). To answer this question, FVB mice and SD rats were exposed to hypoxia up to 3 weeks to identify the ventilatory, hematological, and metabolic changes occurring at whole-body level as well as in mitochondrial O_2_ consumption in the liver (the most metabolically active organ in rodents – accounting for up to 50% of resting O_2_ consumption in mice and rats, Wang et al., 2012; Kummitha et al., 2014). We found that mice have a biphasic increase of minute ventilation, peaking after 7 days of exposure to hypoxia and later increased blood hemoglobin content and hematocrit level. Surprisingly, metabolic rate was higher after 7 days of hypoxic exposure, in line with increased mitochondrial O_2_ consumption in the liver, mediated by the increased activity of complex II of the electron transport chain (ETC). Contrastingly, apart from an excessive increase in hematological parameters, rats show only minor ventilatory or metabolic adjustments.

## Materials and Methods

### Animals

We used male adult (7 weeks old) FVB-NJ mice (Jackson) and Sprague-Dawley rats (Charles River). Animals were housed at the animal care facility at the “Centre de Recherche de l’Institut Universitaire de Cardiologie et de Pneumologie de Québec” (CRIUCPQ) with food and water ad libitum and exposed to 12:12 h light:dark cycles. All experimental procedures were in concordance with the Canadian Council of animal Care. The animal study was reviewed and approved by the Animal Protection Committee of Université Laval, Québec, Canada.

### Exposure to Hypoxia

Animals were exposed to hypoxia (12% O_2_) for 6 h (*n*_mice, rats_ = 15, 13), 1 (*n*_mice, rats_ = 12, 12), 7 (*n*_mice, rats_ = 8, 9), or 21 (*n*_mice, rats_ = 10, 7) days. For the 6 h group, we directly used the plethysmography chambers (see below), while for the other groups, we used plexiglass chambers, controlling oxygen levels with an oxycycler (Biospherix, Redfield, NY). Hypoxic chambers were open once a week during 1 h for changing animals’ cages and replacing water and food. Normoxic controls (*n*_mice, rats_ = 14, 15) were kept in regular room conditions (21% O_2_).

### Ventilatory and Metabolic Parameters

For the last hours of the exposure protocol, animals were quickly transferred to plethysmography chambers and recordings were made for 3 h (while animals were sleeping) using a whole-body plethysmograph (EMKA Technologies, Paris, France). Mice and rats’ plethysmography chambers were constantly supplied with a mix of 12% O_2_ balanced in N_2_, or room air (21% O_2_) for normoxic controls. Inflow was set at 350 ml/min for mice and 1,150 ml/min for rats. A subsampling pump (100 ml/min) was used to control outflow. O_2_, CO_2_, and water pressure levels were continuously measured using specific gas analyzers (OXZILLA, CA-10, RH-300, Sable Systems, Las Vegas, NV; and CD-3A, AEI Technologies, San Francisco, CA). Levels of O_2_, CO_2_, and water pressure in the inflowing air were measured at the beginning, after 1 h, and at the end of the recordings to calculate the oxygen consumption (VO_2_) and CO_2_ production (VCO_2_). Animals were weighed after the recordings.

### Hematological Parameters

After plethysmography recordings, animals were anesthetized and euthanized. Blood samples were taken to measure the hemoglobin (Hb) concentration and hematocrit (Ht). We used an automated spectroscopic system (HemoCue, Ängelholm, Sweden) to measure the concentration of Hb. The Ht was calculated by conventional methods using a dedicated blood capillaries centrifuge.

### Mitochondrial O_2_ Consumption

#### Tissue Sampling

Liver tissue samples (approximately 2 mg) were extracted from rats and mice under normoxia (Nx) or exposed to 1, 7, or 21 days of hypoxia (12% O_2_). Samples were immediately transferred to ice-cold respirometry medium MiR05 (0.5 mM EGTA, 3 mM MgCl_2_*6H_2_O, 60 mM K-lactobionate, 20 mM taurine, 10 mM KH_2_P0_4_, 20 mM HEPES, 110 mM sucrose, and 1 g/L bovine serum albumin pH = 7) supplemented with saponin (50 μg/L) for permeabilization of the cell membrane as previously described (Arias-Reyes et al., 2019).

#### Oxygen Consumption Rates

Quickly after extraction, liver samples were transferred inside the respirometry chambers of the Oxygraph-2k (OROBOROS Instruments – Innsbruck, Austria) filled with the same MiR05 medium supplemented with saponin, and left incubating for 15 min. The mass-specific (pmol O_2_·s^−1^·mg ww^−1^) oxygen consumption rates (OCRs) were measured according to the protocols described below.

In each experiment, the mitochondrial ETC was fueled-up through different electron-transfer pathways by the addition of specific mitochondrial substrates and inhibitors (Gnaiger et al., 2019). The N (NADH electrons through complex I), S (FADH_2_ electrons through complex II), and F (fatty acids oxidation electrons through ubiquinone) pathways were triggered independently or combined to reach spcific mitochondrial activation states namely LEAK and electron transfer (ET). LEAK state results from the proton slippage across the inner mitochondrial membrane toward mitochondrial matrix in presence of mitochondrial metabolic substrates but in absence of ADP. ET state occurs when the electron transfer and oxygen consumption are uncoupled from the adenosine triphosphate (ATP) synthesis by the addition of a protonophore (rapidly returns protons into the mitochondrial matrix independently of the regulation of the ATP synthase) in presence of metabolic mitochondrial substrates (Gnaiger et al., 2019).

### LEAK – ET Protocol

After the 15 min incubation, injections of 5 mM pyruvate and 2 mM malate were added consecutively to the respiratory chambers containing the samples to trigger the N pathway and reach LEAK state. In following, 2.5 mM ADP, 10 μM cytochrome C (see rationale below), and 20 μM CCCP (protonophore) were added to uncouple mitochondria and consequently initiate the ET state. Then, timely injections of 10 mM glutamate, 50 mM succinate, 0.5 mM octanoylcarnitine, and 0.5 μM rotenone were made to record OCRs for N, NS, NSF, and S pathways correspondingly (Doerrier et al., 2018). Finally, 2.5 μM antimycin A (inhibitor of complex III) was added to stop mitochondrial respiration and make corrections for non-mitochondrial OCR. Cytochrome C was used to control the integrity of the outer mitochondrial membrane, thus, if OCR increased more than 20% after the addition of cytochrome C the sample was considered damaged and discarded.

#### ETC Maximum Capacity and Flux Control Ratios

The ETC maximum capacity is defined as the mitochondrial OCR when all the electron-transfer pathways relevant for the analyzed tissue are active during the ET state. In our experiments, the ETC maximum capacity was measured by triggering the NSF pathway. Then, the flux control ratio (FCR) was calculated for each sample by dividing the value of OCR for the electron-transfer pathways N, S, and NS by the value of ETC maximum capacity (NSF pathway). FCR values have the advantages of internal normalization of mitochondrial content (Gnaiger et al., 2019), and of representing the fraction of the ETC maximum capacity covered by each of the N, S, or NS pathways. In this manuscript, FCR is referred as the level of activation of each electron-transfer pathway.

### Activity of Cytochrome C Oxidase (Complex IV)

At the end of the experiments of mitochondrial respiration, after antimycin A injections, 2 mM ascorbate (keeps TMPD in reduced state) and 0.5 mM N,N,N′,N′-Tetramethyl-p-phenylenediamine dihydrochloride (TMPD – specific electron donor to complex IV), were added to measure the OCR exclusive to complex IV. Finally, 100 mM sodium azide (inhibitor of complex IV) was added. The activity of cytochrome C oxidase was calculated by subtracting the background chemical oxidation of the medium (after the addition of azide) from the OCR after the injection of TMPD.

### Data and Statistical Analysis

Plethysmography recordings were analyzed with the software Spike2 v.7.19 (Cambridge Electronic Design). We used averaged values of three different segments along the recording where the breathing pattern was stable at resting values to obtain respiratory frequency (*f*_R_), minute ventilation (V_E_), tidal volume (V_T_), the oxygen consumption (VO_2_), and CO_2_ production (VCO_2_) rates. V_T_ was calculated as described previously (Marcouiller et al., 2014; Ballot et al., 2015; Boukari et al., 2016; Jochmans-Lemoine et al., 2016). VO_2_ and VCO_2_ were calculated by indirect calorimetry as described in (Boukari et al., 2016). Values of *f*_R_, V_T_, VO_2_, and VCO_2_ were allometrically adjusted to make them comparable between species as described previously (Stahl, 1967; Jochmans-Lemoine et al., 2015).

Ventilatory, metabolic, and hematological data were analyzed by two-way ANOVA tests, followed by *post hoc* analysis (Fisher’s Least Significant Difference) using GraphPad Prism 8.3. We used species (mice and rats), and exposure (Nx; 6 h, 1, 7, and 21 days of hypoxia) as factors. Values of V_E_, hematocrit, and VO_2_, were correlated against each other using Spearman’s correlations.

Mitochondrial data were captured using the software DatLab v.7.4.0.4 (OROBOROS Instruments – Innsbruck, Austria). Data was analyzed by two-way RM ANOVA tests using species (mice and rats), and exposure (normoxia; 1, 7, and 21 days of hypoxia) as factors. The combination of mitochondrial respiratory states and active electron-transfer pathways (i.e., LEAK, ET-N, ET-S, ET-NS, and ET-NSF) were the repeated measures. When significant differences in treatments or interactions were found, a *post hoc* Fisher’s Least Significant Difference multiple comparison test was performed.

For all the analyses, test significances were set to *p* < 0.05. Data are presented as means ± SD unless stated differently.

## Results

### Ventilatory Response

#### Mice Have a Stronger Ventilatory Response Than Rats During the Process of Acclimatization to Hypoxia

**Figure 1:**
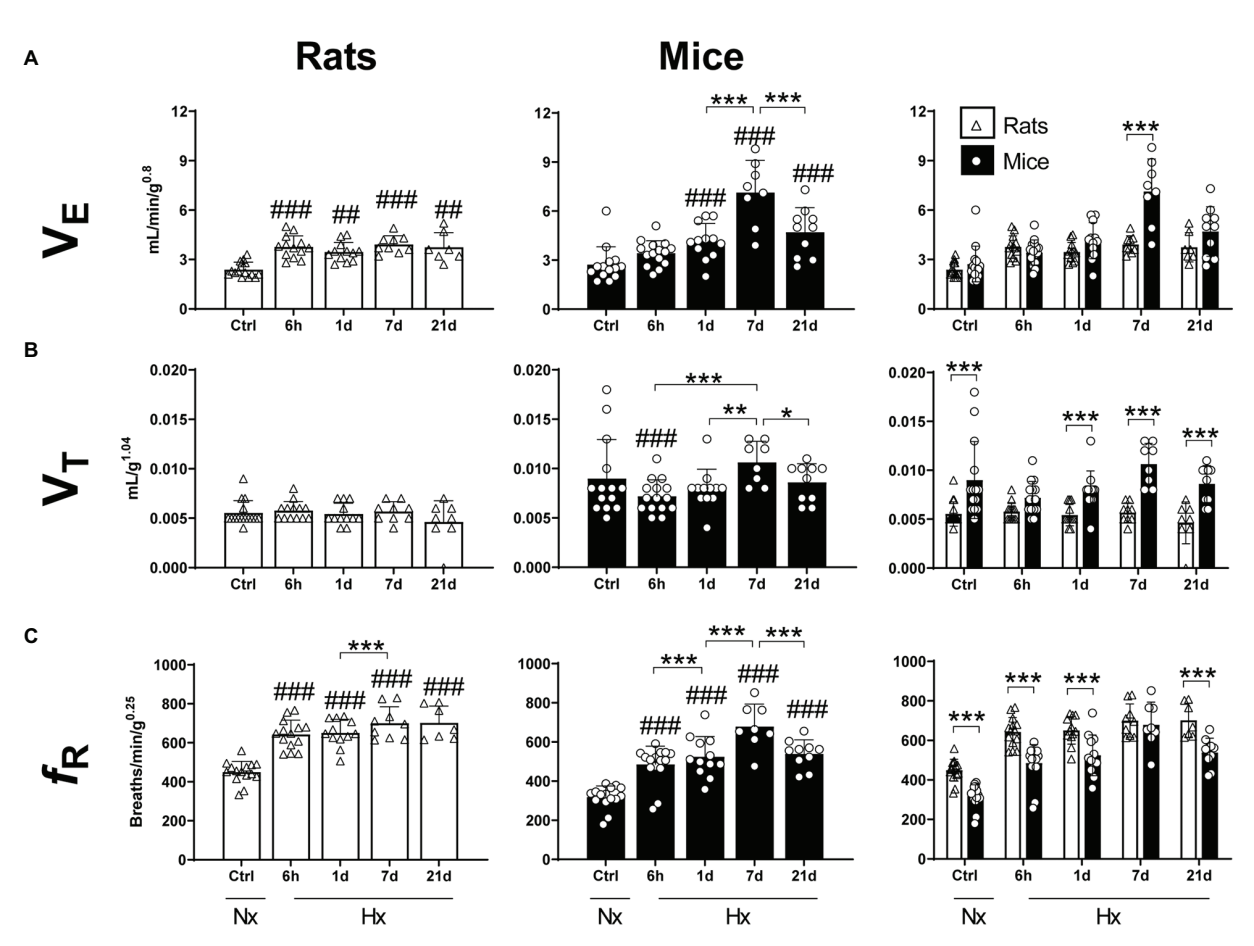
Mass-corrected ventilatory parameters of adult rats and mice in normoxia (Nx) or exposed to 6 h, 1, 7, or 21 days of hypoxia (Hx). Mice show a stronger ventilatory response in hypoxic conditions than rats. **(A)** Mass-corrected ventilation (V_E_), **(B)** mass-corrected tidal volume (V_T_), and **(C)** mass-corrected respiratory frequency (*f*_R_). ^*^p < 0.05; ^**^p < 0.01; and ^***^p < 0.001; ^##^*p* < 0.01 and ^###^*p* < 0.001 vs. the control. *n* = 7–15.

A significant effect of the hypoxic exposure on ventilation was observed in both species (Two-way ANOVA *F*_exposure_ = 25.17; d.f. = 4, 105; *p* < 0.0001; *F*_interaction_ = 9.05; d.f. = 4, 105; *p* < 0.0001). In mice, V_E_ remained unchanged after 6 h of hypoxia in comparison to control levels (Fisher’s LSD test *p* = 0.0608). However, significant increases were observed after 1, 7, and 21 days of hypoxia (Fisher’s LSD test *p*_1d_ = 0.0004; *p*_7d_ < 0.0001; and *p*_21d_ < 0.0001). A peak of V_E_ in mice was reached at day 7 (3-fold normoxic control levels) followed by a significant reduction at day 21 (Fisher’s LSD test *p*_7d vs21d_ < 0.0001; Figure 1A – middle panel). In rats, V_E_ levels increased after 6 h of hypoxia in comparison to control conditions and remained elevated at 1, 7, and 21 days (Fisher’s LSD test *p*_6h_ = 0.0004; *p*_1d_ = 0.0062; *p*_7d_ = 0.0004; and *p*_21d_ = 0.0035; Figure 1A – left panel). Comparing both species, mice showed significantly higher ventilation levels than rats after 7 days of hypoxia (Fisher’s LSD test *p* < 0.0001). However, V_E_ was not different for mice compared to rats in normoxia, 6 h, 1 and 21 days of hypoxia (Fisher’s LSD test *p*_6h_ = 0.35; *p*_1d_ = 0.09; and *p*_21d_ = 0.053; Figure 1A – right panel).

Hypoxia affected V_T_ of mice but not rats (Two-way ANOVA *F*_exposure_ = 2.57; d.f. = 4, 106; *p* = 0.068; *F*_species_ = 71.55; d.f. = 14, 106; *p* < 0.0001; and *F*_interaction_ = 2.473; d.f. = 4, 106; *p* = 0.0488; Figure 1B – left and middle panels). Compared to normoxic controls, mice exposed to 6-h hypoxia showed a significant decrease in VT values (Fisher’s LSD test *p* = 0.0185). Consistently, mice had higher V_T_ values in comparison to rats in normoxia and 1-, 7-, and 21-days hypoxia (Fisher’s LSD test *p*_Ctrl_ < 0.0001; *p*_1d_ < 0.005; *p*_7d_ < 0.0001; and *p*_21d_ < 0.0001). Such difference was not significant after 6 h-hypoxia (Fisher’s LSD test *p* = 0.0649; Figure 1B – right panel).

Hypoxia significantly affected the respiratory frequency of mice and rats (Two-way ANOVA *F*_exposure_ = 49.50; d.f. = 4, 107 *p* < 0.0001; *F*_species_ = 60.56; d.f. = 1, 107 *p* < 0.0001; and *F*_interaction_ = 2.27; d.f. = 4, 107 *p* = 0.067). In mice, hypoxia triggered a significant and progressive increase in respiratory frequency after 6 h, reaching peak values after 7 days of exposure (Fisher’s LSD test *p*_6h_ < 0.0001; *p*_1d_ < 0.0001; *p*_7d_ < 0.0001; and *p*_21d_ < 0.0001). *f*_R_ values dropped by day 21 of hypoxia (*p*_7dvs21d_ = 0.0004). In comparison to control levels, rats showed an augmentation in *f*_R_ after 6 h of hypoxia (Fisher’s LSD test *p* < 0.0001) and maintained elevated values until 21 days of hypoxic exposure inclusively. *f*_R_ values in mice were significantly lower than in rats in either normoxic conditions (*p* < 0.0001), or after 6 h, 1 and 21 days of hypoxia (Fisher’s LSD test *p*_6h_ < 0.0001; *p*_1d_ < 0.0001; *p*_21d_ < 0.0001). Such difference disappears transiently after 7 days of hypoxia (Fisher’s LSD test *p* = 0.574; Figure 1C).

### Hematological Response

#### Mice Have a Late Hematological Response Compared to Rats During the Process of Acclimatization to Hypoxia

**Figure 2:**
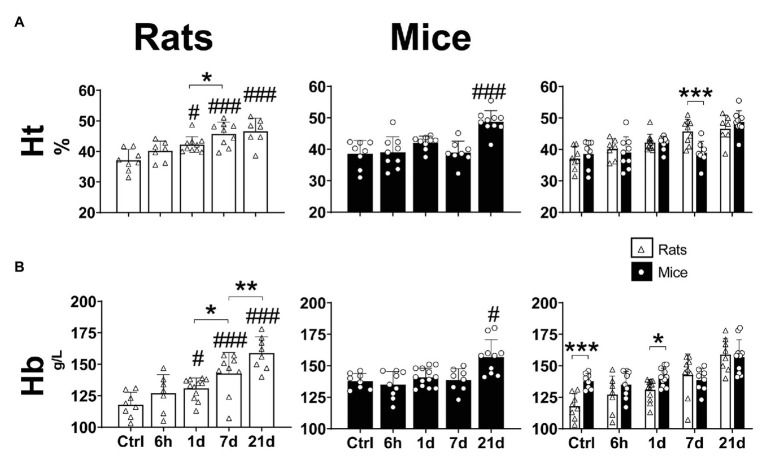
Hematological parameters (hematocrit – **A** and hemoglobin concentration – **B**) of adult rats and mice in normoxia (Nx) or exposed to 6 h, 1, 7 or 21 days of hypoxia (Hx). The hematological response to chronic hypoxia is delayed in mice compared to rats. ^*^p < 0.05; ^**^p < 0.01; and ^***^p < 0.001; ^#^*p* < 0.05 and ^###^*p* < 0.001 vs. the control. *n* = 7–15.

Hypoxia significantly affected the hematocrit (Two-way ANOVA *F*_interaction_ = 3.828; d.f. = 4, 75; *p* = 0.0069) and hemoglobin concentration (Two-way ANOVA *F*_interaction_ = 3.208; d.f. = 4, 82; *p* = 0.017) of mice and rats. The Ht of mice significantly increased in comparison to controls after 21-days hypoxia (Fisher’s LSD test *p* < 0.0001; Figure 2A – center panel). In rats, a progressive augmentation of Ht was observed early after 1 day of hypoxia (Fisher’s LSD test *p* = 0.0046; Figure 2A – left panel). When comparing both species, no differences were observed in normoxic conditions, 6 h, 1 and 21 days of hypoxia. At day 7 of hypoxia, however, mice showed significantly lower Ht levels than rats (Fisher’s LSD test *p* = 0.0003; Figure 2A – right panel).

While the concentration of hemoglobin was significantly increased in mice only after 21 days of hypoxia (Fisher’s LSD test *p* = 0.0046; Figure 2B – center panel), in rats we observed a progressive augmentation after 1 day of hypoxic exposure, reaching maximum values at day 21 (Fisher’s LSD test vs. control: *p*_1d_ = 0.013; *p*_7d_ < 0.0001; *p*_21d_ < 0.0001; *p*_1dvs7d_ = 0.018; and *p*_7dvs21d_ = 0.004; Figure 2B – left panel). In normoxic conditions mice showed significantly higher Hb than rats (Two-way ANOVA *F* = 7.286; d.f. = 1, 82; *p* = 0.008), however, when exposed to hypoxia, no differences between species were observed after 6 h, 7 and 21 days. Moreover, in animals exposed to hypoxia for 1 day, Hb was higher in mice than in rats (Fisher’s LSD test *p* = 0.028; Figure 2B – right panel).

### Metabolic Response

#### Mice and Rats Have a Transitory Drop in Body Temperature During the Process of Acclimatization to Hypoxia

Hypoxia had a significant effect on body temperature (BT) in mice and rats (Two-way ANOVA *F*_exposure_ = 2.712; d.f. = 4, 117; *p* = 0.033; *F*_species_ = 3.466; d.f. = 1, 117; *p* = 0.065; and *F*_interaction_ = 2.517; d.f. = 4, 117; *p* = 0.045). In mice, a significant drop occurred after 1-day hypoxia (Fisher’s LSD test *p* = 0.014), however, after 7 days, control levels were restored. Rats showed an early decline of BT after 6 h of hypoxia (Fisher’s LSD test *p* = 0.01), moreover, control levels were re-established after 7 days. Both species showed similar body temperatures in normoxic conditions, 6-h and 21-days hypoxia. At days 1 and 7 of hypoxic exposure, mice had significantly lower values of BT than rats (Fisher’s LSD test *p*_1d_ = 0.029; *p*_7d_ = 0.016; Table 1).

**Table 1:** Ventilatory, hematological, and metabolic parameters in rats and mice during the process of acclimatization to hypoxia at sea-level and at high-altitude.

	Sea level					High altitude^*^
Rats	Normoxia	Hypoxia 6 h	1 day	7 days	21 days	3,600 masl
Body mass (g)	309.7 ± 88.2	279.8 ± 47.3	242.7 ± 19.7	317.5 ± 31.1	372.6 ± 83.9	232 ± 9
Body temp. (°C)	34.5 ± 1	33.6 ± 1.1	34.3 ± 0.8	34.8 ± 0.7	34.1 ± 0.8	35.2 ± 0.2
V_E_ (ml/min/100 g)	73.3 ± 11.9	115.6 ± 19.7	109.4 ± 19	116.9 ± 16.4	106.3 ± 25.1	153 ± 97
V_T_ (ml/100 g)	0.675 ± 0.153	0.723 ± 0.101	0.665 ± 0.13	0.708 ± 0.099	0.677 ± 0.114	0.79 ± 0.34
*f*_R_ (bpm)	110.2 ± 13.5	157.9 ± 19.5	165.7 ± 16.8	166.3 ± 20.5	155.6 ± 18	186 ± 38
Hematocrit (%)	37.1 ± 3.6	40.1 ± 3.2	42.2 ± 2.6	45.7 ± 3.9	46.6 ± 4.3	60.5 ± 1.1
Hemoglobin (g/L)	117.9 ± 9.9	127 ± 14.8	130.8 ± 8.3	142.8 ± 16.7	158.9 ± 12.9	204 ± 3
VO_2_ (ml/min/100 g)	1.72 ± 0.44	1.59 ± 0.71	2.04 ± 0.69	1.51 ± 0.36	1.54 ± 0.33	1.7 ± 0.26
VCO_2_ (ml/min/100 g)	1.63 ± 0.32	1.17 ± 0.26	1.74 ± 0.33	1.58 ± 0.18	1.25 ± 0.3	1.98 ± 0.41
RER (VCO_2_/VO_2_)	0.95 ± 0.22	0.84 ± 0.35	0.94 ± 0.3	1.1 ± 0.3	0.81 ± 0.09	1.128 ± 0.105
V_E_/VO_2_	46.13 ± 17.18	65.82 ± 25.43	56.98 ± 12.49	80.8 ± 18.82	70.33 ± 17.27	87.7 ± 47.6
O_2_ extraction	12.56 ± 3.63	17.24 ± 8.51	17.61 ± 3.72	12.92 ± 3.28	14.85 ± 3.44	6.7 ± 2.8
**Mice**	**Normoxia**	**Hypoxia 6 h**	**1 day**	**7 days**	**21 days**	**3,600 masl**
Body mass (g)	25.9 ± 2.6	25.1 ± 3.4	24.7 ± 1.1	16 ± 11.9	24.8 ± 1.5	13.7 ± 0.9
Body temp. (°C)	34.3 ± 1.2	33.9 ± 0.9	33.4 ± 0.5	33.7 ± 1.1	34.4 ± 1.1	35.2 ± 0.2
V_E_ (ml/min/100 g)	138.5 ± 54.7	174.3 ± 36.8	211 ± 56.9	367.3 ± 102.2	238.8 ± 77.2	461.4 ± 199.5
V_T_ (ml/100 g)	1.014 ± 0.451	0.824 ± 0.192	0.904 ± 0.219	1.186 ± 0.24	0.978 ± 0.221	1.49 ± 0.67
*f*_R_ (bpm)	141.1 ± 23.7	216.1 ± 37	235.4 ± 46.1	306.6 ± 49.1	240.5 ± 32.9	320.9 ± 75.8
Hematocrit (%)	38.5 ± 4.3	39 ± 5	42.1 ± 2.1	39 ± 3.7	48.8 ± 3.5	47.0 ± 1.7
Hemoglobin (g/L)	137.9 ± 6.1	135 ± 10.6	140.8 ± 7.3	138.8 ± 8.9	156.9 ± 13.8	116 ± 10
VO_2_ (ml/min/100 g)	4.11 ± 1.3	3.56 ± 1.13	3.87 ± 0.98	4.99 ± 0.91	4.85 ± 1.11	5.85 ± 0.96
VCO_2_ (ml/min/100 g)	3.16 ± 0.9	3 ± 0.54	3.18 ± 0.56	4.54 ± 0.9	3.89 ± 1.22	4.95 ± 0.92
RER (VCO_2_/VO_2_)	0.78 ± 0.09	0.93 ± 0.38	0.86 ± 0.23	0.91 ± 0.04	0.8 ± 0.12	0.859 ± 0.169
V_E_/VO_2_	36.22 ± 17.37	52.49 ± 15.9	58.62 ± 24.39	73.34 ± 15.2	50.4 ± 14.33	79.8 ± 31.6
O_2_ extraction	17.62 ± 7.21	18.62 ± 4.55	18.02 ± 7.25	13.12 ± 3.28	18.61 ± 6.14	7.1 ± 3.2

Values are means ± SD. Data for normoxia (Nx) and sea level hypoxia (6 h, 1, 7, and 21 days) is presented in this study.

* High altitude values were published by Jochmans-Lemoine et al. (2015).

#### Mice but Not Rats Increase Their Whole-Body O_2_ Consumption and CO_2_ Production During the Process of Acclimatization to Hypoxia

**Figure 3:**
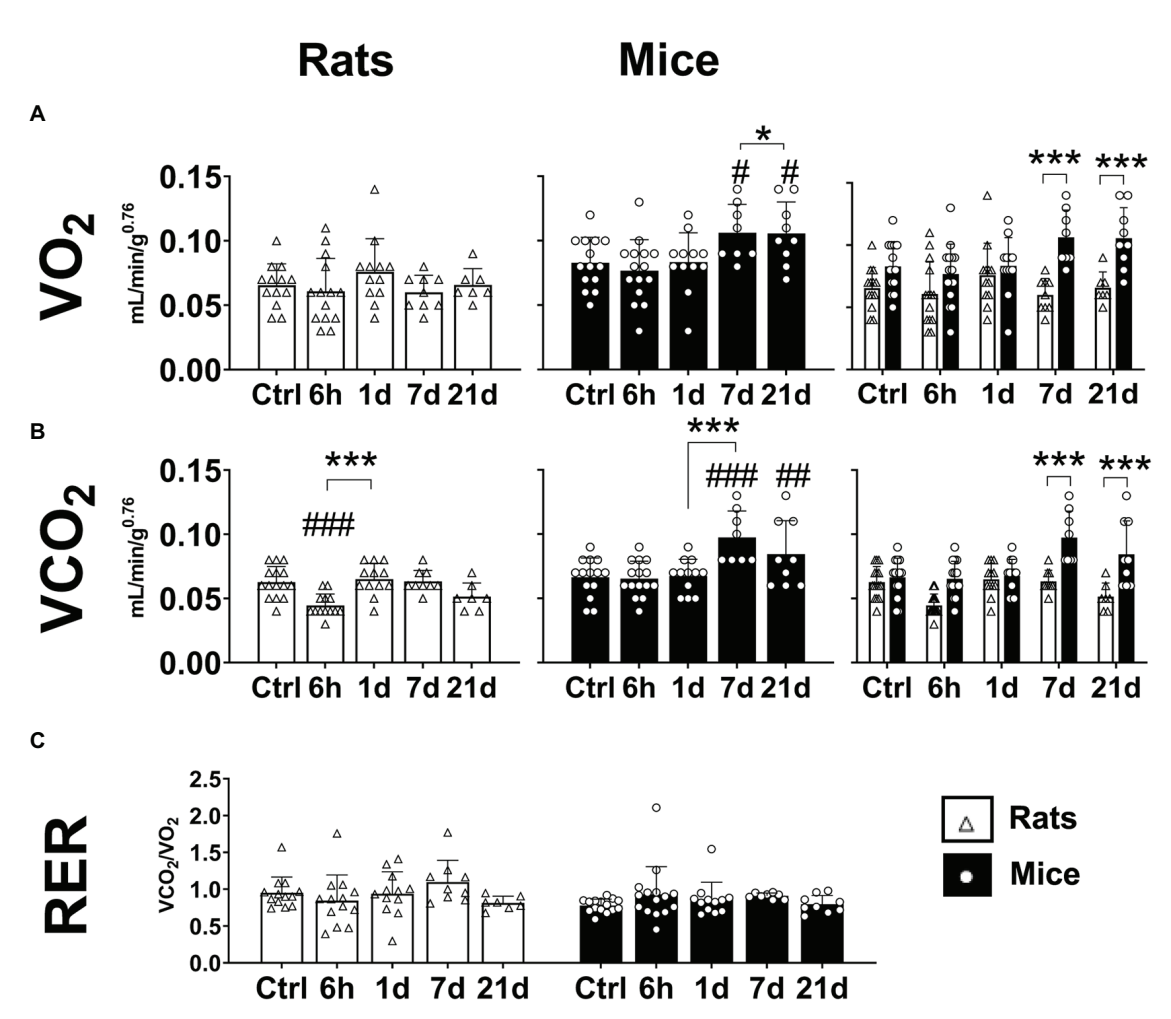
Metabolic parameters of adult rats and mice in normoxia (Nx) or exposed to 6 h, 1, 7, or 21 days of hypoxia (Hx). Mice, but not rats, have an increase in metabolic rate during the process of acclimatization to hypoxia. Mice show increased values of mass-corrected oxygen consumption (VO_2_; **A**) and mass-corrected CO_2_ production **(B)** under hypoxia. The respiratory exchange ratio (RER) remains unchanged in mice and rats **(C)**. ^*^p < 0.05 and ^***^p < 0.001; ^#^*p* < 0.05; ^##^*p* < 0.01; and ^###^*p* < 0.001 vs. the control. *n* = 7–15.

A significant effect of hypoxia on the oxygen consumption (VO_2_) was observed in mice but not in rats (Two-way ANOVA *F*_exposure_ = 2.222 d.f. = 4, 103; *p* = 0.072; *F*_species_ = 36.27 d.f. = 1, 103; *p* < 0.0001; and *F*_interaction_ = 2.865 d.f. = 4, 103; *p* = 0.027). In comparison to normoxic controls, mice exposed to hypoxia for 7 and 21 days showed an increase of 10 and 25% in VO_2_, respectively (Fisher’s LSD test *p*_7d_ = 0.017; *p*_21d_ = 0.016; Figure 3A – center panel). No changes were observed in rats (Figure 3A – left panel). Compared to rats, VO_2_ in mice resulted significantly higher in normoxia and after 7 and 21 days of hypoxia (Fisher’s LSD test *p*_7d_ < 0.0001; *p*_21d_ < 0.0001; Figure 3A – right panel).

CO_2_ production was significantly affected by hypoxic exposure in mice and rats (Two-way ANOVA *F*_exposure_ = 8.375; d.f. = 4, 104; *p* < 0.0001; *F*_species_ = 45.29; d.f. = 1, 104; *p* < 0.0001; and *F*_interaction_ = 5.836; d.f. = 4, 104; *p* = 0.0003). Mice showed an augmentation in VCO_2_ starting after 7 days of hypoxia (Fisher’s LSD test *p*_7d_ < 0.0001; *p*_21d_ < 0.004; Figure 3B – center panel). In rats, a transient reduction of VCO_2_ after 6 h of hypoxia was observed (Fisher’s LSD test *p* < 0.001), however, in rats exposed to hypoxia for 1, 7, and 21 days, VCO_2_ values were not different from normoxic controls (Figure 3B – left panel). When both species were compared, mice showed higher VCO_2_ values than rats after 7 and 21 days of hypoxia (Fisher’s LSD test *p*_7d_ < 0.0001; *p*_21d_ < 0.0001; Figure 3B – right panel).

The respiratory exchange ratio (RER) is calculated as the ratio of VCO_2_ divided by VO_2_. The RER is associated with the favored metabolic fuel (carbohydrates, proteins, or fatty acids). A value close to 1 represents a predominant glycolytic metabolism, while a value closer to 0.7 implies a metabolism more dependent on the oxidation of fatty acids (Deuster and Heled, 2008). In mice, we calculated values of RER between 0.778 and 0.929, while for rats between 0.813 and 1.096, however, no significant effects of hypoxia or species were found (Figure 3C; *F*_exposure_ = 0.25; d.f. = 4, 103; *p* = 0.248; *F*_species_ = 2.256; d.f. = 1, 103; *p* = 0.136; and *F*_interaction_ = 1.212; d.f. = 4, 103; *p* = 0.3104).

### Plasticity in the S (Complex II-Linked) and NS (Complex I&II-Linked) Pathways Upregulates the ETC Maximum Capacity During Acclimatization to Hypoxia in Liver Mitochondria of Mice but Not in Rats

**Figure 4:**
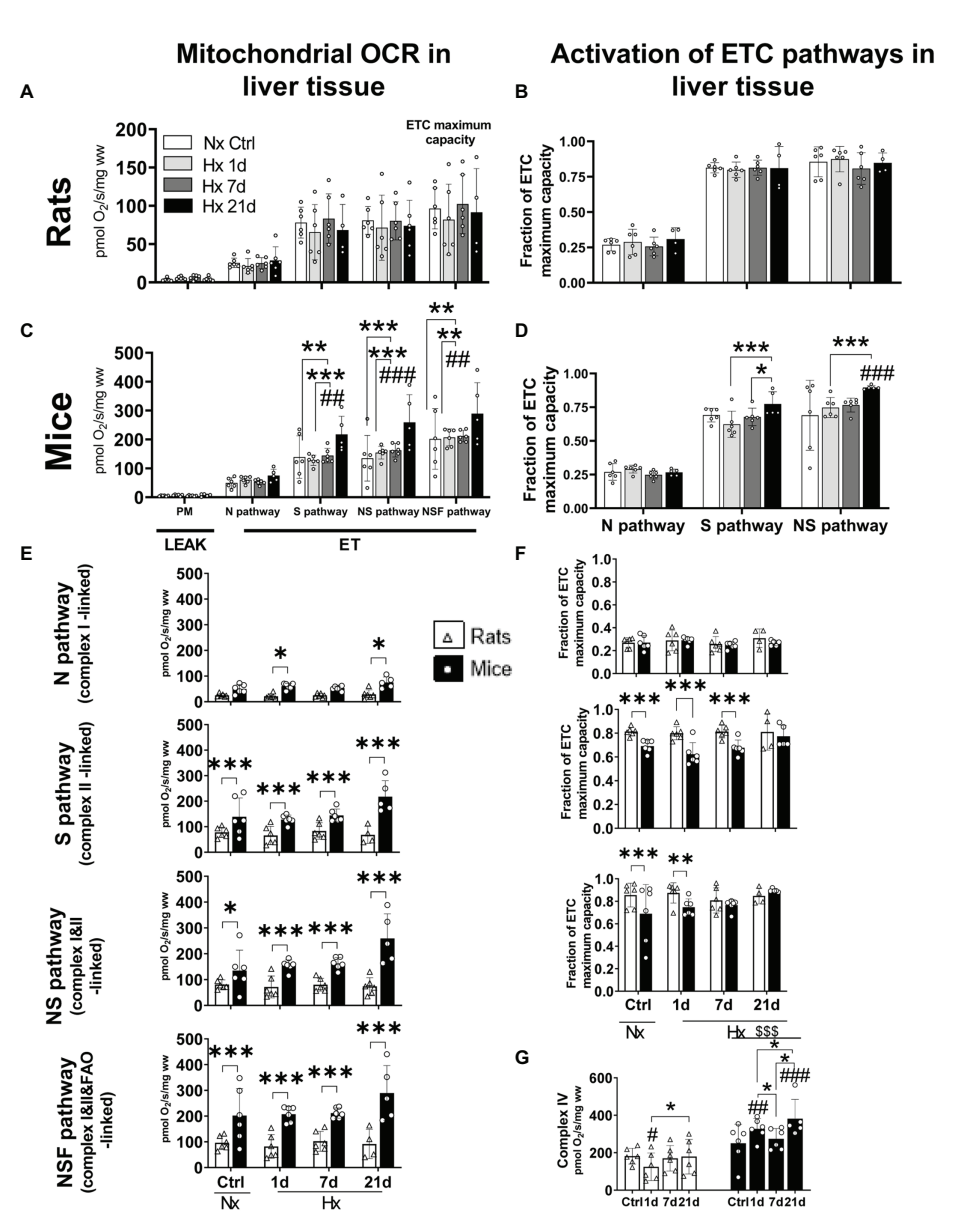
Oxygen consumption rates (OCR) and activation of mitochondrial electron transport chain (ETC) pathways in liver tissue of adult rats and mice exposed to normoxia (Nx) or 1, 7, or 21 days of hypoxia (Hx). The OCR was measured during inactive adenosine triphosphate (ATP) production (LEAK) or uncoupled electron transfer (ET) fueled with substrates for the N (complex I), S (complex II), NS (complexes I&II), or NSF (complexes I&II and fatty acids oxidation) pathways. Activation values represent the fraction of the ETC maximum capacity covered by the activation of N (complex I-linked), S (complex II-linked), or NS (complexes I&II) pathways. OCR in rats’ liver-mitochondria is not affected by hypoxia **(A)**. Likewise, rats showed no changes in the activation of N, S, or NS pathways in normoxic nor hypoxic conditions **(B)**. Mice increased its OCR (+44%) after 21-days hypoxia due to an overactivation of S pathway (complex II). Additionally, the maximum ETC capacity (NSF pathway) was 70% increased after 21 days of hypoxia **(C)**. A non-significant overactivation (+11%) of S pathway occurred in mice after 21 days of hypoxia together with the increased activation of NS pathway (+20%; **D**). Mice have higher OCR levels than rats in normoxic and hypoxic conditions. This occurs mainly because of higher S pathway (complex II) activities. The ETC maximum capacity in mice is higher in normoxia (+52%) and hypoxia (up to +69% at day 21) than in rats **(E)**. Rats show a higher activation of N, S, and NS mitochondrial pathways compared to mice during acclimatization to hypoxia prior 7 days of hypoxia. The overactivation of S pathway in mice compensates for these differences after 21-days hypoxia **(F)**. The overactivation of complex IV triggered by hypoxia hints to an improved efficiency in oxygen use in mice but not in rats **(G)**. ^*^p < 0.05; ^**^p < 0.01; and ^***^p < 0.001; ^#^*p* < 0.05; ^##^*p* < 0.01; and ^###^*p* < 0.001 vs. the control. *n* = 4–6.

We tested the effect of hypoxia on the oxidative machinery in the ETC of liver mitochondria of mice and rats during LEAK and ET states. Furthermore, we calculated the FCR as a measurement of the fraction of the ETC maximum capacity covered by the activation of N, S, or NS electron-transfer pathways. No changes were observed for LEAK state in any species either in normoxia or hypoxia (Figures 4A,C). Regarding ET state, in mice, while the N pathway (complex I-linked) remained unaltered in normoxia and hypoxia, after 21-days hypoxia the S (complex II-linked), NS (complexes I&II-linked), and NSF (complexes I&II&FAO-linked) pathways increased significantly in comparison to normoxic control levels (RM ANOVA Fisher’s LSD test *p*_S_ = 0.0002; *p*_NS_ < 0.0001; *p*_NSF_ < 0.0001; Figure 4C). Accordingly, the activation of NS pathway was upregulated by 30% after 21 days of hypoxia (RM ANOVA Fisher’s LSD test *p* < 0.0001). The activation of S pathway was seemingly increased as well (+12%) after 21-days hypoxia, and while the difference with the normoxic controls was not statistically significant (RM ANOVA Fisher’s LSD test *p* = 0.0548), the improved activation was significant in comparison to 1 and 7-days hypoxia levels (RM ANOVA Fisher’s LSD test *p*_1d_ < 0.0005; *p*_7d_ < 0.0259; Figure 4D). Rats showed no alterations in oxygen consumption nor in the activation of electron-transfer pathways during ET state (Figures 4A,B). When both species were compared, mice showed higher OCRs during ET state than rats in both normoxic and hypoxic conditions (RM ANOVA *F*_species_x_exposure_ = 10.42; d.f. = 7, 39; *p* < 0.0001), independently of the activation of S (Fisher’s LSD test *p*_ctrl_ = 0.023; *p*_1d_ = 0.0018; *p*_7d_ = 0.0023; and *p*_21d_ < 0.0001), NS (Fisher’s LSD test *p*_ctrl_ = 0.027; *p*_1d_ < 0.001; *p*_7d_ < 0.001; and *p*_21d_ < 0.0001), or NSF (Fisher’s LSD test *p*_ctrl_ < 0.0001; *p*_1d_ < 0.0001; *p*_7d_ < 0.0001; and *p*_21d_ < 0.0001) pathways. The same occurred when only the N pathway was active after 1 and 21 days of hypoxia, however, no differences were evidenced in normoxic control conditions and after 7-days hypoxia (Fisher’s LSD test *p*_ctrl_ = 0.2216; *p*_1d_ = 0.0486; *p*_7d_ = 0.1649; and *p*_21d_ = 0.0253; Figure 4E). Rats and mice showed similar levels of activation (FCR) of the N pathway in normoxic and hypoxic conditions, however, the S pathway was significantly less active in mice than in rats when exposed to normoxia, 1 and 7-days hypoxia (*p*_1d_ < 0.0001; *p*_7d_ < 0.001). This difference disappears after 21 days of hypoxia (*p* = 0.438). Similarly, the activation of NS pathway was weaker in mice compared to rats in normoxic conditions and after 1 day of hypoxia (*p*_ctrl_ < 0.0001; *p*_1d_ = 0.002). Differences disappear after 7 and 21 days of hypoxia (*p*_7d_ = 0.303; *p*_21d_ = 0.315; Figure 4F). Finally, we measured the activity of complex IV, the final enzyme in the oxidation of oxygen, because it is known to be structurally modified upon exposure to hypoxia. In this regard, mice and rats were not different in normoxic conditions, however, when under hypoxia, mice reached higher values independently of the time of exposure (Fisher’s LSD test *p*_ctrl_ = 0.005; *p*_1d_ < 0.0001; *p*_7d_ < 0.0001; and *p*_21d_ < 0.0001). Mice significantly increased the activity of complex IV after 1 and 21 days-hypoxia in comparison to the normoxic group and showed control-like values at day 7 (Fisher’s LSD test *p*_1d_ = 0.002; *p*_7d_ = 0.329; and *p*_21d_ < 0.0001). In contrast, rats had a transient decline at day 1, then re-establishing normoxic control levels (Fisher’s LSD test *p*_1d_ = 0.0194; *p*_7d_ = 0.636; and *p*_21d_ = 0.9161; Figure 4G).

**Figure 5:**
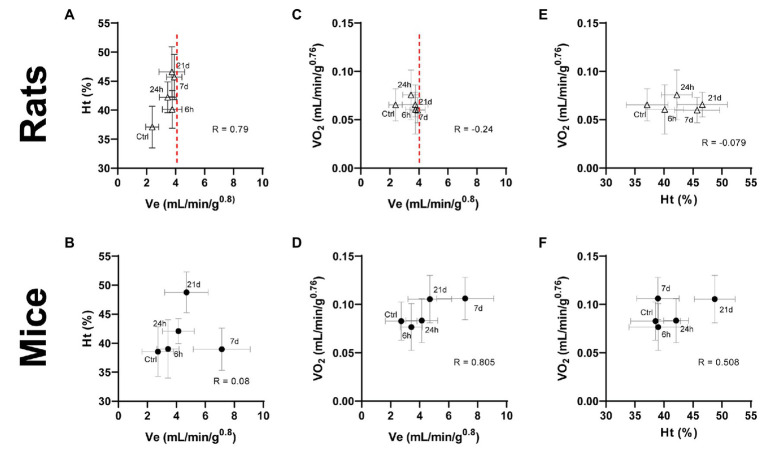
Interaction between ventilatory and hematological parameters **(A,B)** and the effect of ventilatory **(C,D)** and hematological **(E,F)** parameters on the whole-body oxygen consumption in adult rats and mice exposed to normoxia (Ctrl) or 1, 7, or 21 days of hypoxia. A metabolic increase (oxygen consumption – VO_2_) in response to chronic hypoxia in mice is supported by cooperative changes in ventilatory and hematological systems. This outcome in rats is impaired due to a limited ventilatory response evident after 6 h of hypoxia (dashed line), thus, requiring excessive hematological compensations to cope with hypoxic conditions.

## Discussion

Our data show that mice and rats have divergent profiles of ventilatory, hematological, metabolic, and mitochondrial acclimatization to hypoxia. At ventilatory level, mice have an early and progressive augmentation in respiratory frequency and minute ventilation, while this response is limited in rats. On the other hand, the increase of hematocrit and hemoglobin occurs only after 3 weeks of hypoxic exposure in mice. Rats on the contrary, increased both hematological parameters during the first day of hypoxia. Furthermore, while there is an elevation of whole-body oxygen consumption and CO_2_ production in mice, metabolism in rats remains unaffected. These changes correspond well with the increased activity of the electron-transfer pathways involving complex II (S pathway) in liver mitochondria of mice, which is absent in rats.

### Ventilatory Adjustments in Mice Are More Beneficial Than in Rats

We observed in FVB mice and SD rats, a rapid increase (after 6-h and 1-day hypoxia correspondingly) in respiratory frequency and ventilation, in agreement with previous observations in our laboratory (Jochmans-Lemoine et al., 2016). This response is well-known to occur in mammals and birds (Storz et al., 2010) since hyperventilation contributes to limit the decrease in alveolar oxygen partial pressure, thus supports oxygen diffusion toward blood. In mice, such increase continued for 7 days, reaching values of V_E_ 2.6-fold and *f*_R_ 2.1-fold control levels. Similar sustained increasing trends in ventilation after different times of exposure to hypoxia have been observed in other species like the deer mice, pekin duck, and bar-headed goose (Black and Tenney, 1980; Powell, 2007; Ivy and Scott, 2017). After 21 days of hypoxia, however, mice presented an attenuation in V_E_ and *f*_R_, a change hypothesized to be advantageous for species living permanently under chronic hypoxia (i.e., high altitude) by reducing the oxygen cost of breathing when other adaptations are present (Powell, 2007). At this point, both parameters were only 1.7 control levels and were about 30% lower than the values reported for FVB mice born and grown at 3,600 m of altitude (Jochmans-Lemoine et al., 2015). In rats, V_E_ and *f*_R_ values were sustained around 1.6-fold control levels starting after 6 h and remained unchanged even at 21 days of hypoxia. Interestingly, the values of mass-corrected V_E_, we observed in rats after 6-h, 1-, 7-, and 21-days hypoxia (*V*_E6h_ = 3.4 ± 0.8; *V*_E1d_ = 3.4 ± 0.6; *V*_E7d_ = 3.9 ± 0.5; and *V*_E21d_ = 3.7 ± 0.9 ml/min/g^0.8^), were only 15–20% lower than those reported by Jochmans-Lemoine et al. (2015) in adult (2–3 months old) laboratory rats raised at high-altitude (4.5 ± 2.7 ml/min/g^0.8^), suggesting that the process of ventilatory acclimatization to hypoxia in rats is somehow limited to the acute phase of exposure (6 h). This divergent ventilatory response between mice and rats may be explained by the higher protein level of HIF-1α, in the brainstem (the region of the brain that contains the respiratory control network) after 6 h of hypoxic exposure (O_2_ 12%) observed in mice but not in rats (Jochmans-Lemoine et al., 2016). Contrary to our findings, Schmitt et al. (1994) reported a continuous increase in V_E_ until day 14 of hypoxic exposure in male SD rats, although, animals were kept under lower (10%) oxygen concentration, and it is well known that this affects the magnitude of the hypoxic ventilatory response (Powell et al., 1998). Finally, despite the changes observed in V_E_ and *f*_R_, V_T_ were only slightly affected by hypoxia in mice and not affected at all in rats. Moreover, V_T_ in mice was significantly higher than in rats in normoxia and hypoxia, as we reported previously (Jochmans-Lemoine et al., 2015). Overall, these results show that mice have a more plastic respiratory control system than rats.

### Hematological Acclimatization Is Stronger and Quicker in Rats Than Mice

Increases in hematocrit and hemoglobin concentration are common hematological responses to hypoxic exposure in lowland individuals (Bullard, 1972; Monge and Whittembury, 1976; Monge and León-Velarde, 2003), helping to ensure the oxygen availability to supply tissues and organs. In this study, we found significantly increased hematocrit and hemoglobin levels after 21 days of hypoxia (12% O_2_) in comparison to normoxic controls in mice (Ht = +26%, Hb = +14%) and rats (Ht = +26%, Hb = +35%). Interestingly, in mice, values of Ht observed after 21 days of hypoxia were identical to those reported previously for adult mice from high-altitude colonies (cf. Table 1). However, Hb levels in mice were higher than those reported at high altitude (Jochmans-Lemoine et al., 2015). It has been argued that the increase in Hb levels is classical in lowland native species because mammals native to high altitude shows unchanged Hb levels upon hypoxic exposure (Monge and Leon-Velarde, 1991). Furthermore, genetic studies in “wild” South American *Mus musculus* collected at sea level and high altitude found no signs of natural selection in the locus coding for subunits alpha and beta of hemoglobin, in fact, both South American populations coincided genetically with European haplotypes, leading to the conclusion that high altitude residency did not affect hemoglobin affinity for oxygen as an adaptive trait in this species (Storz et al., 2007). These observations suggest that the hematological mechanisms of acclimatization to hypoxia in mice are triggered late and are constrained to the first 21 days of hypoxic exposure. On the other hand, rats showed lower values of Ht and Hb in this study than those reported for high-altitude animals (Table 1) hinting that the hematological acclimatization in this species extends longer than 3 weeks. Another possibility is that the phenotype shown by adult rats in high-altitude colonies is also affected by changes during gestation or development. The later was shown to be true in adult SD rats (3 months-old) exposed to postnatal hypoxia (12% O_2_ for 10 days) as newborns. These animals developed identical levels of Ht and Hb concentration to those observed in adult rats grown in high-altitude lab colonies (Lumbroso and Joseph, 2009; Jochmans-Lemoine et al., 2015). A limitation of our study is that we did not evaluate the change in cardiovascular parameters such as heart rate and arterial oxygen saturation (SaO_2_) along the process of acclimatization to hypoxia. It is known that cardiovascular features interact with respiratory and hematological adjustments to counterbalance the diminished oxygen availability. Indeed, the heart rate of SD rats living permanently at high altitude (3,600 masl) is seemingly increased compared to sea level animals. This does not occur in FVB mice (Jochmans-Lemoine et al., 2015).

### Elevated Metabolic Rates in Mice Are Supported by Coordinated Ventilatory and Hematological Adjustments That Are Disjointed in Rats

The metabolism of animals exposed to hypoxia has been classically pointed to be reduced as a strategy to save energy in conditions of limited oxygen availability (Scott et al., 2008; Storz et al., 2010). However, cumulative evidence shows that upon chronic hypoxic exposure metabolic rates can increase in vertebrate species adapted to hypoxic conditions such as the deer mice and the bar-headed geese (Ward et al., 2002; Scott et al., 2008, 2018; Scott and Milsom, 2009; Ivy and Scott, 2017). We found that in rats, hypoxia had no effect on the O_2_ consumption and CO_2_ production rates, while these values increase in mice suggesting an increased metabolic expenditure linked with the mechanisms underlying hypoxia-acclimatization. The values of VO_2_, we found in our experiments after 21 days of hypoxia were about 20% lower than those observed in mice born and grown in high-altitude colonies (see Table 1), suggesting further margins to increase metabolic rates during permanent residency at high altitude. However, body weights after 21 days-hypoxia are higher than in high-altitude animals (see Table 1), such difference may be due to the effects of hypoxia during gestation and growth similar to those we described for SD rats (Lumbroso and Joseph, 2009). Such metabolic upsurge may also be interpreted as a compensatory mechanism to restore the body temperature independently of the hypoxic exposure. However, the metabolic rate (VO_2_) in mice was augmented after day 7 and remained this way until day 21 of hypoxia, while the rectal temperature was significantly lower only at day 1. Interestingly, the VO_2_ values we observed after 21 days of hypoxic exposure in rats are much closer (less than 10%) to those reported for high-altitude born and grown SD rats than they are for the FVB mice (Table 1), suggesting wider margins for plasticity of metabolic systems in mice compared to rats. Moreover, after the rise in oxygen consumption occurring at day 7 of hypoxia, VO_2_ was significantly higher in mice than rats, similarly to what happens in high-altitude acclimatized animals (Hochachka et al., 1983; Ivy and Scott, 2017).

Notably, the forementioned ventilatory and hematological systems normally act in coordination to compensate any imbalance in the relation of oxygen demand and supply (d_O2_/q_O2_) produced by hypoxia. Indeed, we observed increased levels of Ht and Hb in mice only after 21 days of hypoxia, while these changes occurred much earlier in rats. This suggests that in mice the ventilatory compensations are sufficient to supply enough O_2_ during the first 3 weeks. In rats, however, the restricted ventilatory response accompanied by an early and persistent hematological modulation hint to an improper compensation of the imbalanced ratio d_O2_/q_O2_. Consequently, we explored the relations among the response patterns of V_E_, Ht, and VO_2_ by plotting these features against each other. We noticed distinct interactions between ventilatory, hematological, and metabolic parameters in mice and rats. In mice, an initially large ventilatory response is withdrawn after the day 7 of hypoxia in synchrony with the activation of hematological adjustments (Figure 5B). Hematological tuning during acclimatization to hypoxia is classically thought to be triggered slightly later than respiratory modulations, and is considered energetically more efficient than ventilatory and cardiovascular modifications (Monge and León-Velarde, 2003). Moreover, the enhanced ventilation accompanies the increased oxygen consumption up to a steady state reached at day 7 of hypoxic exposure (Figure 5D). The latter is further supported by augmented levels of hematocrit (Figure 5F), suggesting that ventilatory mechanisms act early during the process of acclimatization of mice and are coordinately backed by subsequent hematological adjustments to ensure an enhanced oxygen supply. Rats, however, have limited ventilatory adjustments, hence require stronger hematological compensations to cope with hypoxic conditions. This is well illustrated by a clear threshold in ventilation right after 6 h of hypoxic exposure while (red dashed line in Figure 5), in contrast, continuously rising hematological adjustments are present for over 3 weeks (Figure 5A). Likewise, although no significant statistical correlation was found, negative correlation coefficients indicate that VO_2_ is not further increased either by ventilatory or hematological modifications following the acute response occurring in the first day of hypoxia (Figures 5C,E). The fact, we observed a concurrent acute initiation of ventilatory and hematological mechanisms in rats further supports the hypothesis that blood-level modifications play a more significant role compared to ventilation in the acclimatization of rats.

### Plasticity in Liver Mitochondria of Mice, but Not Rats, Permits a More Efficient Use of Subcellular Oxygen in Hypoxic Conditions

We used liver tissue to study the role of mitochondria in the metabolic response to chronic hypoxia since this organ accounts for around 50% of the whole-body energy expenditure in mice and rats (Wang et al., 2012; Kummitha et al., 2014). We quantified the maximum capacity of the ETC by measuring the OCR uncoupled from the ATP synthesis (Gnaiger et al., 2019). We did not find significant alterations in the mitochondrial oxygen consumption in the liver of rats. Concurring with our observations, Costa et al. (1988) found no changes in the respiration of isolated liver-mitochondria after 9-months hypoxia (equivalent to 4,400 masl) in Wistar rats. Contrastingly, Pickett et al. (1979) observed a slight initial reduction in the activity of complex II during the first 2 weeks of hypoxia with an outburst over normoxic levels around the third week of normobaric hypoxia in SD rats. Of note, the levels of oxygen used in this work were much lower than those we applied to our animals (equivalent to 6,600 vs. 4,000 masl respectively), under these circumstances, the lack of response we found suggests that liver mitochondria in rats may only respond to extreme oxygen limitation.

Interestingly, we did see patterns in the liver mitochondrial respiration of mice that coincide with features of species adapted to high-altitude hypoxia. Deer mice show augmented mitochondrial respiratory capacities in the diaphragm muscle after exposure to hypoxia (equivalent to 4,300 masl for 6–8 weeks; Dawson et al., 2018; Scott et al., 2018). As well, in the gastrocnemius muscle, this species have elevated expression levels and activity of enzymes involved in fatty acid β-oxidation and oxidative phosphorylation (Cheviron et al., 2012), and a higher density of subsarcolemmal mitochondria (Mahalingam et al., 2017). HA native deer mice can maintain high metabolic rates during acclimatization to hypoxia (Cheviron et al., 2012; Ivy and Scott, 2017). This occurs either in field conditions at HA (Hayes, 1989) or in response to cold exposure (Cheviron et al., 2012). These biochemical changes are supported by high capillarity and oxidative fiber density (Lui et al., 2015), and result in elevated mitochondrial respiratory capacities (Mahalingam et al., 2017). In our mice, liver mitochondria show an increased ETC maximum capacity (NSF pathway) after 3-week hypoxia propelled by an increased activity of complex II, while complex I remains unchanged. Similar enhancement of complex II activity in ETC under hypoxic exposure has been reported in brain cortex of mice and hypoxia-resistant rats (Lukyanova, 1997; Cáceda et al., 2001; Lukyanova et al., 2008; Lukyanova and Kirova, 2015). Contrary to complex I, which is sensitive to drops in intracellular oxygen concentration, the activity of complex II is only dependent on the availability of its substrate: succinate (Hawkins et al., 2010). Remarkably, in mammals, succinate is accumulated in mitochondria during hypoxia (Hohl et al., 1987; Cáceda et al., 1997; Lukyanova et al., 2018) and acts as a stabilizer of HIF-1α by attenuating the activity of the polyhydroxylases (PHDs; which mark HIF1-α for denaturation in normoxic conditions), thus supporting acclimatization to hypoxia (Fuhrmann and Brüne, 2017). Also, succinate is involved with auxiliar (albeit less efficient) avenues to produce ATP by (1) substrate phosphorylation (produces GTP) catalyzed by succinate thiokinase and (2) the reduction of oxalacetate to succinate that is coupled with the reverse reaction of malate dehydrogenase and the concomitant electron transfer to the ETC *via* complex I (Cáceda et al., 2001). Complex II (succinate dehydrogenase) participates in the second mechanism. Moreover, complex II is also the gateway for cytosolic NADH to enter mitochondria *via* the glycerophosphate shuttle and for the reduced equivalents from fatty acids oxidation, that way having a great participation in ATP production. Accordingly, the fatty acids metabolism has been suggested to be augmented in high-altitude conditions (León-Velarde and Arregui, 1994). Overall, this supports the notion that an increased CII activity as reported here would promote a more efficient O_2_ utilization by liver mitochondria in mice. Indeed, the increased activity of complex II is regarded as an “*evolutionarily formed, urgent, protective, regulatory, and compensatory mechanism*” occurring in most tissues upon hypoxic exposure and during acclimatization (Lukyanova and Kirova, 2015). Contrastingly, Leon-Velarde et al. (1986) reported no changes in the respiration of isolated liver mitochondria in Swiss mice exposed to hypobaric hypoxia (equivalent to 4,400 masl) for 90–120 days concluding that isolated mitochondria are not suitable to perform experiments on the effects of hypoxia. However, the exposure period they tested is long enough to allow mitochondrial ETC to recover levels of respiratory complexes activity seen in normoxic animals (Lukyanova and Kirova, 2015). Finally, we learned that liver mitochondria in mice consume more oxygen than their counterparts in rats regardless of the availability of oxygen and the active electron-transfer pathways. This may explain, at least in part, the higher whole-body O_2_ consumption in mice compared to rats. Interestingly, during normoxia and the first day of hypoxic exposure, the activation of S and NS pathways is lower in mice than in rats, however, by day 7 of hypoxia such difference in activation disappears hinting to the existence of a “spare capacity” in the liver mitochondrial ETC, a feature observed in other tissues in high-altitude-adapted species (Storz et al., 2010; Lukyanova and Kirova, 2015). Overall, the higher plasticity during acclimatization to hypoxia in mice supports our hypothesis of a genetic background supporting the ability of mice to cope with hypoxia, which is limited or absent in rats.

### Why Mice Respond Better to Chronic Hypoxia Than Rats?

Whereas cellular mechanisms work generally at maximum efficiency, especially in the context of energy generation and expenditure, there are cases when “spare” room for further improved efficiency remains as genetical vestiges from ancient more demanding circumstances. This confers populations the capacity to invade new ecological niches thanks to previously acquired appropriate structural and/or functional changes, with the concomitant emergence of multiple “adaptive peaks” (Griffiths et al., 2000), a process called “pre-adaptation” (Monge and León-Velarde, 2003). Indeed, the acclimatizing mitochondrial traits, we observed in mice have been previously posed as well-adaptive or as distinctive from high-altitude adapted species (Lukyanova, 1997; Lukyanova and Dudchenko, 1999; Cáceda et al., 2001; Czerniczyniec et al., 2015; Lukyanova and Kirova, 2015). On the other hand, acclimatizing changes at systemic level in rats are almost exclusively reliant on hematological adjustments, a strategy that has been tagged as disadvantageous due to its limitations to compensate for the reduced levels of oxygen delivery, and instead causing stress to the cardiovascular system (Lumbroso and Joseph, 2009; Storz et al., 2010; Lumbroso et al., 2012) on top of the inadequate or inexistent ventilatory, metabolic, and mitochondrial responses.

Mice (*Mus musculus*) and rats (*Rattus rattus* and *Rattus norvegicus*) are species with very different natural and phylogeographical histories, this can perfectly justify the physiological differences found in their mechanisms of acclimatization to hypoxia. The phylogeographic origin of *M. musculus* lies in the Irani-, Iraqi-, and Pakistani-Himalayan region, from where they initially colonized European and Asian lands by successfully overcoming migrations across mountain chains facing high-altitude challenges and certainly undergoing processes of natural selection (Bonhomme and Searle, 2012). On the contrary, *R. rattus* originated in India and South-Asia, and dispersed towards lowlands evading the northern mountain passes (Aplin et al., 2011). Even more contrastingly, *R. norvegicus* are original from the lowlands of central China and their expansion occurred almost strictly linked to human migration in recent historic times (Song et al., 2014; Puckett et al., 2016). Together with our present and past (Lumbroso and Joseph, 2009; Lumbroso et al., 2012; Jochmans-Lemoine et al., 2015, 2016, 2018; Jochmans-Lemoine and Joseph, 2018) results, this phylogeographic pattern strongly supports the hypothesis of mice being pre-adapted to high-altitude hypoxia.

We conclude that FVB mice and SD rats develop divergent physiological and mitochondrial mechanisms of acclimatization to hypoxia. Mice have an adaptive response with adjustments in oxygen capture, distribution, and utilization that allow them to maintain an unaltered or even increased whole-body and liver-mitochondrial metabolism. On the contrary, rats deploy an excessive and detrimental alteration in oxygen transport systems in blood and fail to trigger adjuvant ventilatory, metabolic, and subcellular procedures, therefore, presenting a deficient acclimatizing response. The adaptive adjustments during the process of acclimatization of mice may explain, at least in part, their ability to colonize high-altitude habitats in contrast to rats and further supports the hypothesis that mice are pre-adapted to high-altitude hypoxia.

## Data Availability Statement

The raw data supporting the conclusions of this article will be made available by the authors, without undue reservation.

## Ethics Statement

The animal study was reviewed and approved by Animal Protection Committee of Université Laval, Québec, Canada.

## Author Contributions

CA-R performed all the experiments and data analyses. CA-R, JS, and VJ wrote and approved the manuscript. All authors contributed to the article and approved the submitted version.

### Conflict of Interest

The authors declare that the research was conducted in the absence of any commercial or financial relationships that could be construed as a potential conflict of interest.
